# pH-dependent nanodiamonds enhance the mechanical properties of 3D-printed hyaluronic acid nanocomposite hydrogels

**DOI:** 10.1186/s12951-020-00647-w

**Published:** 2020-06-10

**Authors:** Dae Gon Lim, Eunah Kang, Seong Hoon Jeong

**Affiliations:** 1 College of Pharmacy, Dongguk University-Seoul, Gyeonggi, 10326 Republic of Korea; 2 School of Chemical Engineering and Material Science, Chung-Ang University, Seoul, 06974 Republic of Korea

## Abstract

Nanocomposite hydrogels capable of undergoing manufacturing process have recently attracted attention in biomedical applications due to their desired mechanical properties and high functionality. 3D printing nanocomposite hydrogels of hyaluronic acid (HA)/nanodiamond (ND) revealed that the addition of ND with the low weight ratio of 0.02 wt% resulted in higher compressive force and gel breaking point, compared with HA only nanocomposites. These HA nanocomposite hydrogels loaded with surface functionalized ND allowed for the enforced compressive stress to be tuned in a pH-dependent manner. HA nanocomposite hydrogels with ND-OH at pH 8 showed an increase of 1.40-fold (0.02%: 236.18 kPa) and 1.37-fold (0.04%: 616.72 kPa) the compressive stress at the composition of 0.02 wt% and 0.04 wt, respectively, compared to those of ND-COOH (0.02%: 168.31 kPa, 0.04%: 449.59 kPa) at the same pH. Moreover, the compressive stress of HA/ND-OH (0.04 wt%) at pH 8 was mechanically enhanced 1.29-fold, compared to that of HA/ND-OH (0.04 wt%) at pH 7. These results indicate that the tunable buffering environment and interaction with the long chains of HA at the molecular level have a critical role in the dependency of the mechanical properties on pH. Due to the pH stability of the ND-OH nanophase, filament-based processing and layer-based deposition at microscale attained enforced mechanical properties of hydrogel. Fine surface tuning of the inorganic ND nanophase and controlled 3D printing leads to improved control over the pH-dependent mechanical properties of the nanocomposite hydrogels reported herein.
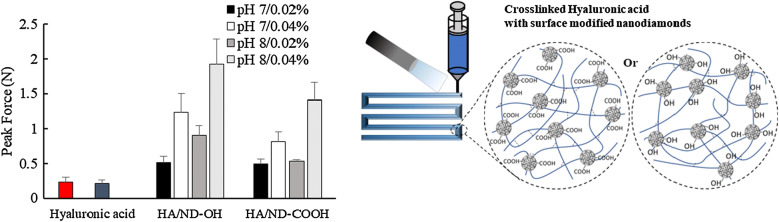

## Introduction

Three-dimensional (3D) printing of biomaterials has gained attention in the pharmaceutics to direct development toward customized therapeutics that increase patient compliance. Computation-based manufacturing processes, including tablets [1], medical devices [2, 3], tissue engineering [4–6], and tumor modeling [7] have benefited from 3D printing. Layer-by-layer-based construction provides exquisite control over parameters such as drug amount, size, multidrug loading, scaffold porosity, and release profiles simply through geometric modification. 3D printing can be classified into several categories including stereolithographic printing by UV irradiation, powder bed/powder jetting system, selective laser sintering of thermoplastic polymer, and extrusion-based system [1, 3]. Processing ink materials has a pivotal role in the choice of mechanical 3D-printing type; the geometric shape and stability of the constructed filaments from the nozzle depend on the ink’s physical and chemical properties, including thermoplasticity, UV curability, and rheological properties.

The functionality of a thermoplastic biodegradable polymer or natural biopolymer with versatile chemical modification has been extensively studied for theranostics and drug delivery. There have been several recent studies exploring the microscopic mechanical properties of these polymers, as well as their cellular functionality and drug delivery [8–13]. In particular, modified nanocomposite hydrogels with enhanced mechanical and functional properties have been beneficial in the fields of tissue engineering, medical devices, and depot formulation. Scalable micro-environments created by 3D printing have allowed for the study of cell proliferation and estimated tumor modeling of breast cancer bone metastasis, with relation to geometric factors [7]. Even the distribution of metallic ions in 3D hydrogel has been shown to amplify the signal intensity of DNA sensing [14]. Mechanically enhanced biomaterials and natural biopolymers have been composited with metal [15], carbon based materials [15–19], glass [20–22], cellulose crystal [23, 24], and clay [25, 26] to form nanocomposites for a variety of applications. Nanocomposite hydrogels that incorporate diverse nanophase inorganic particles are challenging because metastatic materials mimicking biological tissues require a variety of considerations including softness, biocompatibility, strength, and structurally compatible elasticity. The specific functionality granted by nanocomposite hydrogels with inorganic materials would lead to improved electrical conductivity [27, 28], energy absorbance [29–31], as well as cellular [18, 26, 32, 33] and protein interactions [2], in addition to mechanical enhancement.

Hyaluronic acid (HA), is a natural non-immunogenic biopolymer that is ubiquitous in connective tissue. HA has been used for various forms of medicine and long-term release implant in vivo. HA has the advantage that data on safety in the human body are present in large quantities compared to other artificial polymers. In addition, HA is easy to perform chemical modification, which can modify the physicochemical properties [34]. Due to the above advantages, various studies on chemically modifying HA as bioink for 3d printing have been conducted such as pentenoate-functionalized HA [35], β-cyclodextrin/adamantane induced HA [36, 37]. In this study, commonly used crosslinkable methacrylated HA was used for nanocomposite hydrogel with addition of nanodiamonds for 3D processable printing.

**Scheme 1:**
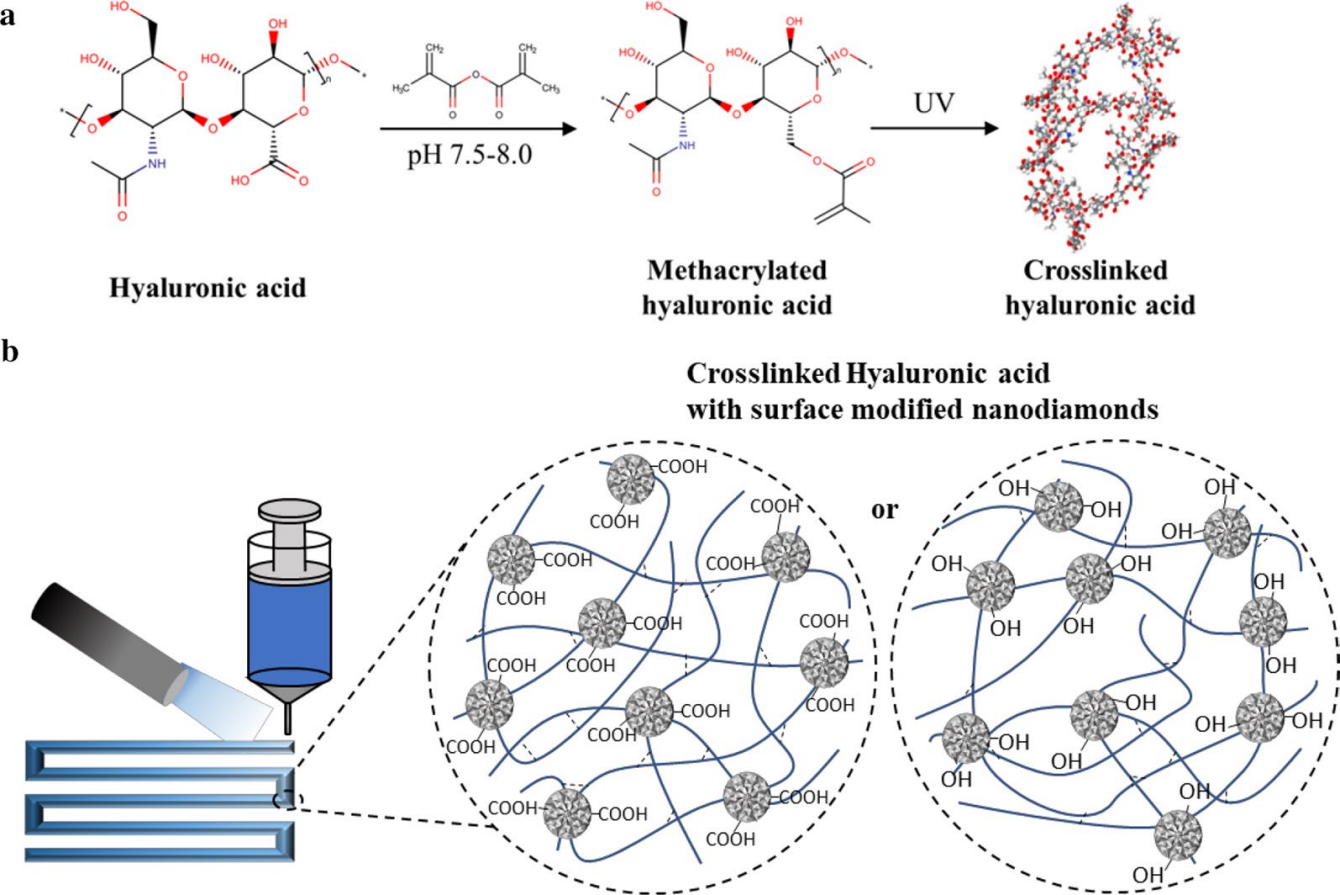
**a** End group modification of UV-crosslinkable methacrylated HA, **b** schematic picture of 3D printing UV-crosslinked HA composited with surface modified NDs

Among the nanoscale carbon based materials, nanodiamonds (NDs) have been chosen due to their hydrophilicity and pH-dependent surface functionality, along with their convenient chemical modality [38–40]. Detonated NDs, of approximately 5 nm, followed by an acid-wash are amenable to a diverse range of surface modifications. Depending on the tunable surface properties, NDs can be created as agglomerates ranged from approximately 5 nm to several hundred nm in size [41]. These 5 nm NDs have been used to deliver small hydrophobic chemical drugs [42] and negatively charged RNA [43], as well as crosslinked for thermoplastic composites [39]. The high surface-to-volume ratio and inert hardness also provided lubrication to reduce friction and wear for even biomaterials, which is an important parameter in the extrusion-based 3D-printing process [44]. Here, nanocomposite hydrogels mixed with UV-crosslinkable HA and surface-modified NDs were processed using a nozzle-based extrusion 3D-printing manufacturing system (Scheme 1b). The rheological properties of nanocomposite hydrogel precursor and mechanically enforced nanocomposite hydrogels were characterized with the addition of carboxylated and hydroxylated NDs at varied pH.

## Experimental methods

Methacrylated hyaluronic acid (MeHA) was synthesized by a substitution reaction between hyaluronic acid (HA) and methacrylic anhydride (MA) in water (Scheme 1a) [45, 46]. HA (1500–2000 kDa range, Xian DN biology, China) was dissolved at 0.5% (w/v) in deionized water (20 g of HA in 4 L of deionized water), and a sixfold molar excess of methacrylic anhydride relative to HA repeat units, was added dropwise to the solution. The pH of reacting mixture was adjusted to 7.5–8.0 with 5 M NaOH solution, and the reaction was continuously stirred overnight at 5 °C with re-adjustment of pH. The reactant was precipitated with a fivefold volumetric excess of cold acetone. White MeHA was separated, rinsed three times with cold acetone, re-dissolved in deionized water, and lyophilized.

Carboxylated NDs and hydroxylated NDs were prepared as reported in previous studies [42, 43]. In brief, the commercial products of carboxylated ND-COOH were supplied by Nanoresource Co., Ltd. (Seoul, Korea). The carboxyl groups of ND-COOH were reduced by the addition of NaBH_4_ to generate hydroxyl end groups on the ND substrates. The ND-COOH (50 mg) was dispersed with sonication and mixed with NaBH_4_ (100 mg) in ethanol (100 mL) in an ice bath. The mixture was stirred for 24 h at 60 °C under reflux and then washed with ethanol (50 mL × 2) and distilled water (50 mL × 3). The final product of ND-OH dispersed in distilled water was lyophilized for further characterization.

Rheological flow properties of HA/ND hydrogel were investigated by a stress-controlled rheometer (Haake Mars IIM, Thermo Electron, Germany) at 25 °C. The parallel-plate fixture was set with a diameter of 25 mm and a 1 mm gap. Before each experimental run, the specimen was kept at the set temperature for 10 min to establish a homogeneous thermal state and eliminate residual thermal history. Viscosity was measured by a rotation ramp method with the shear rate from 0 to 100 (1/s). Oscillation stress sweep tests were carried out at a frequency of 10 Hz in a stress range of 1–100 Pa. In addition, time stress tests were carried out using a constant shear stress and frequency (5.0 Pa and 10 Hz). Frequency sweep tests were carried out at a sheer stress of 5.0 Pa in a frequency range of 1–10 Hz. The results were analyzed using the Haake Rheowin Data Manager 3.6 software.

The HA/ND mixture was prepared at desired concentrations and processed for 3D printing under the following conditions. A micro-dispensing pump machine was used as the extrusion system (Rokit Invivo, Rokit Healthcare, Seoul, Korea), and all printing was conducted at room temperature. During extrusion, a UV precuring system (6 W/cm^2^, 385 nm) near the nozzle was used to cure the extruded filament and construct 3D-layers. The dispensing nozzles were respectively 21 gauge (514 μm inner diameter), 23 gauge (337 μm inner diameter), and 26 gauge (260 μm inner diameter) to deposit precursor filaments. The distance between two adjacent filaments was approximately 1.0 mm and the vertical step of 0.2 mm. The nozzle printing speed was 2 mm/s and the standoff distance was set as 2 mm. After printing, the 3D constructions were cured using UV light (18 W/cm^2^) for 15 min. Digital 3D design programmed in printing path code was performed using 3ds max 2018 (Autodesk Inc., Mill Valley, CA, USA). Printed 3D porous networks of crosslinked hyaluronic acid structure were imaged by scanning electron microscopy (SEM) using an EM-30 SEM (COXEM, Daejeon, Korea) at a 20.0 kV acceleration voltage. Crosslinked HA structures were lyophilized and then sputtered with gold under argon atmosphere using an SPT-20 ion coater (COXEM, Daejeon, Korea).

Compressive mechanical strength of the HA structures was determined by texture analyzer (TA.XT plus, Stable Micro Systems Ltd., UK). The indenter was equipped with a 5 N load cell (resolution of 0.1 mN) and 2 mm diameter flat-ended cylindrical stainless steel indentation probe. Compressive force was applied to the 3D-printed hydrogel vertically and the rate of force measurement was 20 μm/s with a triggering force of 0.001 N as the initial depth of hydrogel.

Load–displacement data from indentation measurement were used to obtain compressive moduli using the mathematical model introduced by Oliver and Pharr [47]. Effective modulus, E^*^, was

$$ \frac{1}{{E^{*} }} = \frac{{1 - \upsilon_{s}^{2} }}{{E_{s} }} + \frac{{1 - \upsilon_{i}^{2} }}{{E_{i} }} $$

where E and $\upupsilon$ are Young’s modulus and Poisson’s ratio, respectively, and the subscripts s and i present specimen and indentor, respectively. Each term indicates elastic displacement resulted from a sample and indentor. However, since the indentor was very rigid compared to the soft specimen (stainless steel vs. soft hydrogel), contribution from the second term can be ignored.

The most widely used mathematical model of indentation for soft tissue can be expressed as following,

$$ E^{*} = \frac{1}{\beta } \times \frac{dP}{dw} \times \frac{1}{2} \times \frac{\sqrt \pi }{A} = S/2a $$

where $$ \upbeta $$ is a geometric constant such that $$ \upbeta = 1 $$ for flat ended indentation probe and $$ {\raise0.7ex\hbox{${dP}$} \!\mathord{\left/ {\vphantom {{dP} {dw}}}\right.\kern-0pt} \!\lower0.7ex\hbox{${dw}$}} $$ is the stiffness (S) at instantaneous displacement $$ (w)\,{\text{and}}\,{\text{load }}\left( P \right) $$. Derivatives ($$ {\raise0.7ex\hbox{${dP}$} \!\mathord{\left/ {\vphantom {{dP} {dw}}}\right.\kern-0pt} \!\lower0.7ex\hbox{${dw}$}} $$) of incorporation with load and instantaneous displacement is used to account for the non-linearity of soft tissue. Measured stiffness at each measurement was calculated to obtain a calibrated effective modulus.

## Results and discussion

**Fig. 1:**
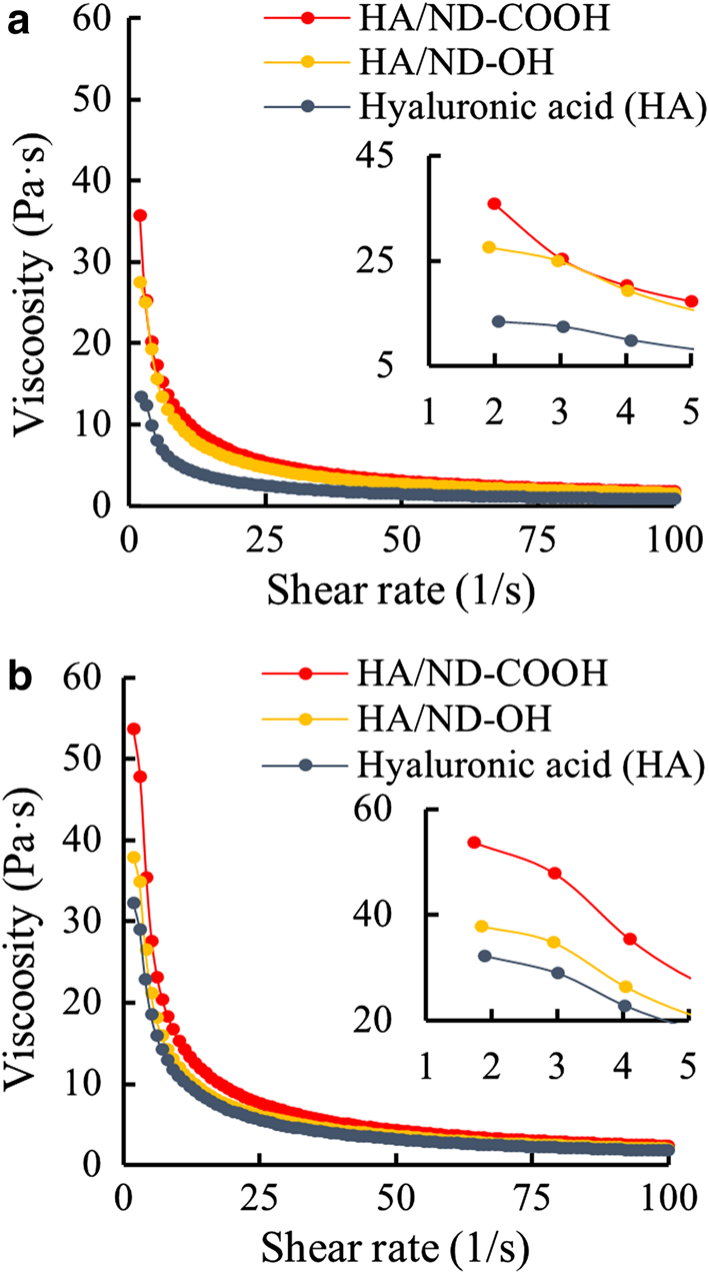
Viscosity vs. shear rate of HA nanocomposite hydrogel precursor composited with ND-OH or ND-COOH at **a** pH 7 and **b** pH 8

The rheological properties of hydrogel precursor of HA/ND were determined with viscosity change and shear rate. To prepare nanocomposite hydrogel precursors, 0.02 wt% carboxylated or hydroxyl ND were mixed with 2 wt% of MeHA at pH 7 and pH 8. After the full pre-sheared state of the nanocomposite hydrogel precursor, the shear rate decreased significantly and the viscosity change is recorded during the subsequent 100 s. As shown in Fig. 1, the shear-thinning effect of all HA, HA/ND-COOH, and HA/ND-OH nanocomposite hydrogel precursor generated low viscosities (approximately 2 Pa s) in the pre-sheared zone. After the shear rate was decreased and fluids were fully structured, the viscosity of the nanocomposite hydrogel precursors increased rapidly with a stiff gradient to approximately 33.24 Pa s on average in 2/s. The viscosity of the nanocomposite hydrogel precursors with nanodiamonds was 35.74 Pa s for ND-COOH and 27.5 Pa s for ND-OH at pH 7, respectively. In addition, the viscosity of MeHA was 13.34 Pa s at pH 7 and 32.29 Pa s in 2/s. It is noted that nanocomposite HA hydrogel containing NDs showed higher viscosity and shorter response time, compared to HA only hydrogels, indicating a pH controllable filament shape in the 3D-printing process. The nanocomposite HA hydrogel precursors showed higher viscosity at pH 8, compared to those at pH 7. Rheological characteristics such as viscosity may vary depending on the degree of polymerization of HA, but by using ND, it was possible to form a viscosity suitable for printing despite the use of a lower content of HA. Compared to the results obtained by measuring the viscosity using a commercially available product (Cellink^®^) and pentenoate-functionalized HA, viscosity in 2/s was 52.8 Pa s for HA/ND-COOH (at pH 8), about 100 Pa s for Cellink^®^, and 120 Pa s for 3% pentanoate functionalized HA [35]. Compared to the results of other studies, the viscosity was relatively low, but concentration of hyaluronic acid (2 wt%) was lower than other hydrogel (at least 3%), and HA/ND hydrogel showed stronger shear thinning effect than other materials. As the nanodiamond was added, the increased viscosity increased up to 270% compared to when HA was used alone, but it was confirmed that the viscosity decreased very rapidly with increasing shear rate. HA/ND-COOH (at pH 8) showed the highest viscosity 15.33 Pa at 10/s, which is a 70% decreased value from initial viscosity. Weak shear-thinning originates from high molecular weight hyaluronic acid itself [48], but nanodiamonds appear to induce shear-thinning more strongly. Shear-thinning hydrogels were researched by blending shear-thinning materials to hyaluronic acid [13, 36] and nanodiamonds performed a similar role in hyaluronic acid. As the negatively charged end groups of HA and ND increased, electrostatic interaction between nanophase ND and HA resulted in the augmented viscosity, which was further influenced by the 3D-printing manufacturing process.

The change of zeta potential and hydrodynamic diameter of ND-OH and ND-COOH agglutinates depending on pH were fully characterized in previous study [42]. In brief, the zeta potentials of ND-COOH were negative − 22.5 ± 0.2 mV and − 27.0 ± 0.4 mV, at pH 7 and pH 8, respectively. ND-COOH dispersion showed the decrease in zeta potential as the increased pH from 7 to 8 due to deprotonation of carboxyl groups. ND-OH produced by the reduction of ND-COOH showed stable zeta potentials in the wide pH ranges, ranged from − 51.4 ± 0.9 to − 44.5 ± 0.7 mV at the pH 4–10. The zeta potentials of ND-COOH and ND-OH were negative zeta potential of − 22.5 ± 0.2 mV and − 42.7 ± 1.2 mV at pH 7, respectively, suggesting good colloidal stability. The diameters of the dispersed ND-OH and ND-COOH agglutinate were also affected by environmental pH. Depending on the pH, the average sizes of ND-COOH were changed into 105.2 nm at pH 7 and 77.9 ± 1.6 nm at pH 8. The average sizes of ND-OH were 61.5 ± 0.3 nm at pH 7 to 67.2 ± 3.3 nm at pH 8, showing less pH sensitivity and high dispersion stability. These pH dependence by surface functional group. pH dependent size changes of ND agglutinates are closely related with the dissociation constants (pKa) of carboxylic acids 5.0 and the alcohol 14–16, as reported. The degree of ionization might determine colloidal stability and resulting agglutinate size due to interparticle interaction.

Regarding with these, pH dependent colloidal stability and negative zeta potentials of ND-COOH and ND-OH might have repulsive electrostatic interaction between negative HA. The agglutinate size resulted from environmental pH dependent charge effect might also contribute the significantly increased shear thinning effect. Considering that size of ND-COOH became significantly increased at pH 7, increased shear thinning effect should be considered with colloidal size. ND-OH with smaller stabilized particle size and higher negative zeta potentials showed less shear thinning effect, compared to the ND-COOH. Resulting shear thinning effect by addition of NDs might be complex of factors including colloidal size, surface charge, surface functionality. The resulting higher shear thinning effect by addition of ND, compared to HA only gained the processible high quality of printing ink.

**Fig. 2:**
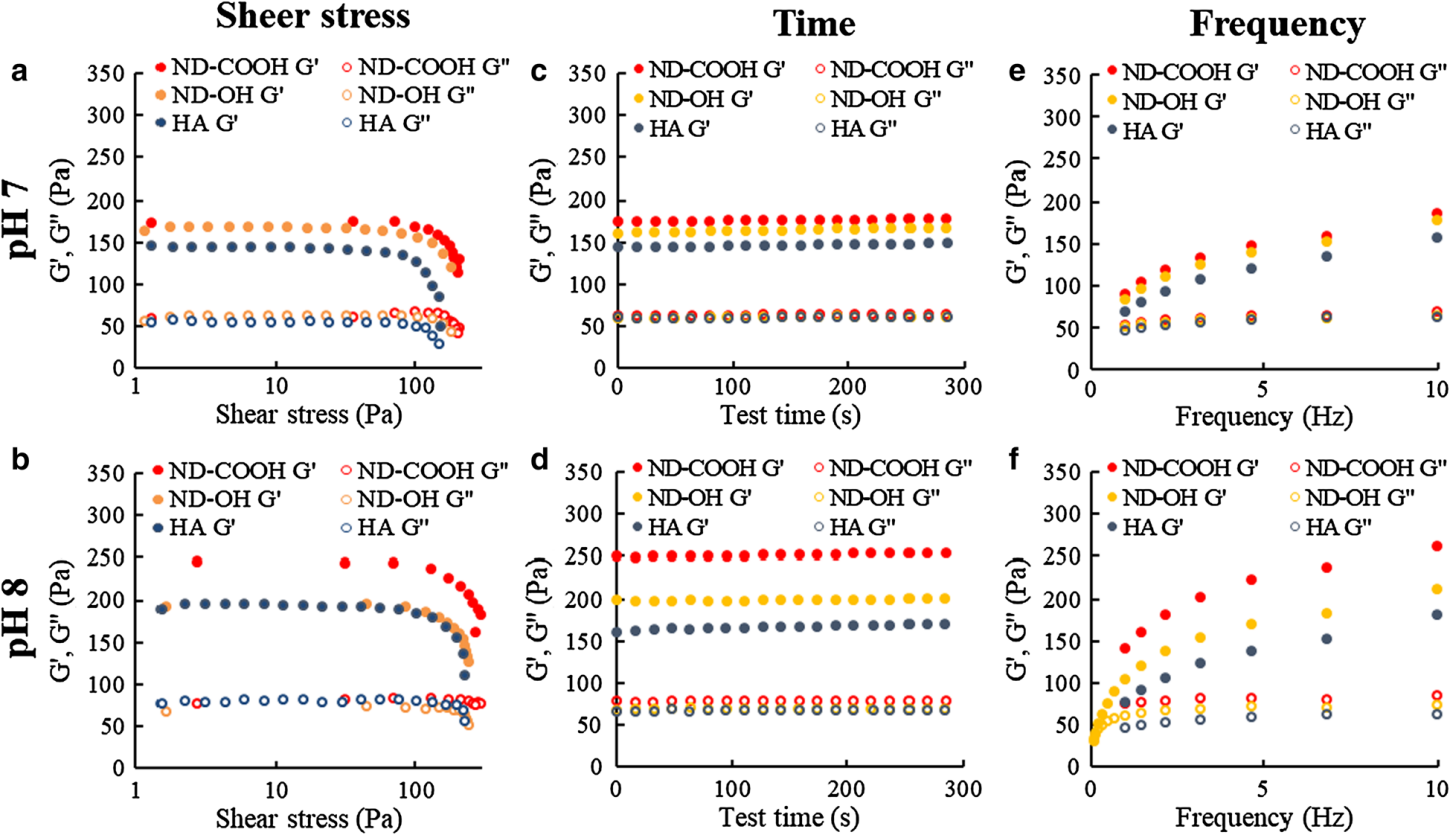
Rheological properties and relaxation behavior of nanocomposite hydrogel precursor of HA/ND. Storage modulus G′ and Loss Modulus G″ of HA and HA/ND hydrogel precursor mixed with 0.02 wt% as function of shear stress at **a** pH 7 and **b** pH 8. Storage modulus G′ and Loss Modulus G″ of HA and HA/ND as a function of time at **c** pH 7 and **d** pH 8. Storage modulus G′ and Loss Modulus G″ of HA and HA/ND as a function of frequency at **e** pH 7 and **f** pH 8

Shear thinning was observed using nanocomposite hydrogel precursors, which can be attributed to the electrostatic interactions from the COO– and hydrogen bonding (–OH) between ND surface and HA. The rheological properties of the nanocomposite hydrogel precursors estimated as storage modulus G′ and loss modulus G″ revealed that the HA/ND-COOH and HA/ND-OH hydrogel increased by 1.18 (185.4 Pa) and 1.13 (177.6 Pa) fold (G′), 1.10 (67.86 Pa) and 1.02 (63.24 Pa) fold (G′′) at pH 7, respectively, compared to HA only hydrogel at 62.83 rad/s (10 Hz) (Fig. 2a). This result suggests that the existence of physical interaction, i.e., electrostatic interaction between –COO– on the ND surface and HA chains, contributes to the increase in G′ and G″ in HA/ND hydrogel precursors. Remarkably, G′ and G″ in HA/ND-COOH hydrogel precursor at pH 8 was increased 1.4-fold (260.5 Pa) and 1.26-fold (85.26 Pa), respectively, compared with those of HA/ND-COOH hydrogel at pH 7. This proves that the deprotonated ND-COOH surface became hydrophilic and was interacting with the HA chains. In a pH dependent manner, the shear-thinning behavior of the HA/ND hydrogels precursor occurred through quick absorption and desorption of HA chains from ND surface, affecting the 3D printing structure and filament stability.

**Fig. 3:**
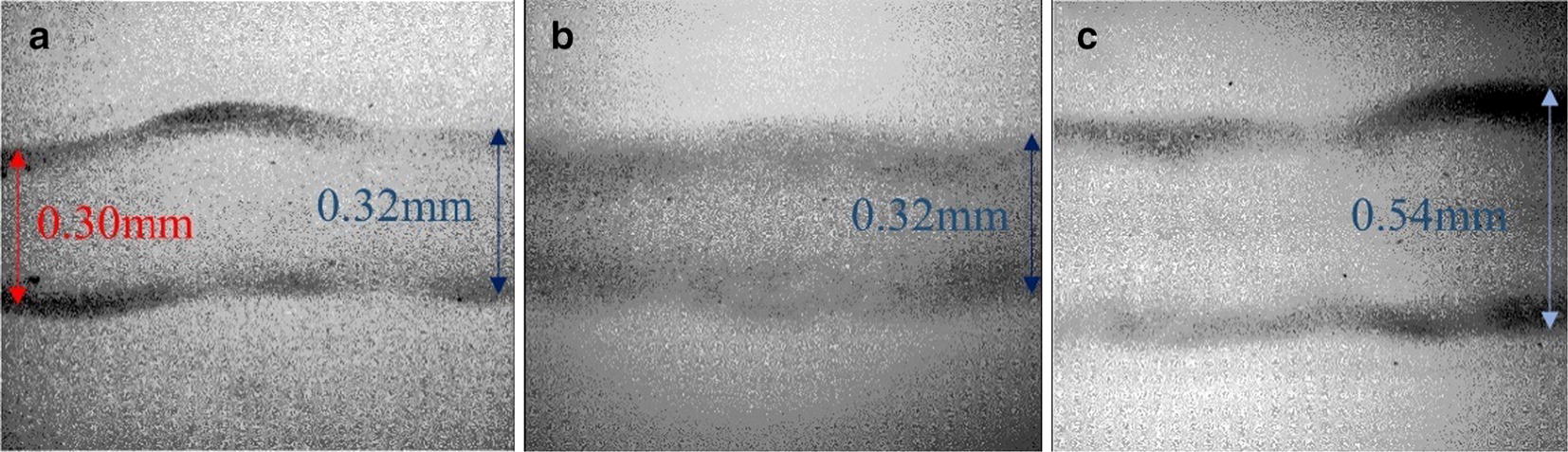
Filament sizes for the HA/ND-OH nanocomposite hydrogel precursor with varied needle size under the same printing parameter configurations (moving speed of 2 mm/s). Needle size: **a** 26 gauge, **b** 23 gauge, **c** 21 gauge

The filament diameter is generally controlled by operating parameters including the inner diameter of the needle, nozzle path speed, and standoff distance on the filament width. When deposited as a liquid filament of nanocomposite hydrogel precursor, filament structure and stability are critical during the extrusion. In general, filament widths can be controlled by varying the pressure of the cylinder, the inner diameter of the nozzle, the path speed, and the standoff distances [11, 49]. A 26 gauge needle was used to extrude stable filaments of the HA/ND-COOH and HA/ND-OH hydrogel nanocomposites, demonstrated in Fig. 3. The same operating conditions were used to explore the dependence of mechanical properties on pH between the different NDs.

**Fig. 4:**
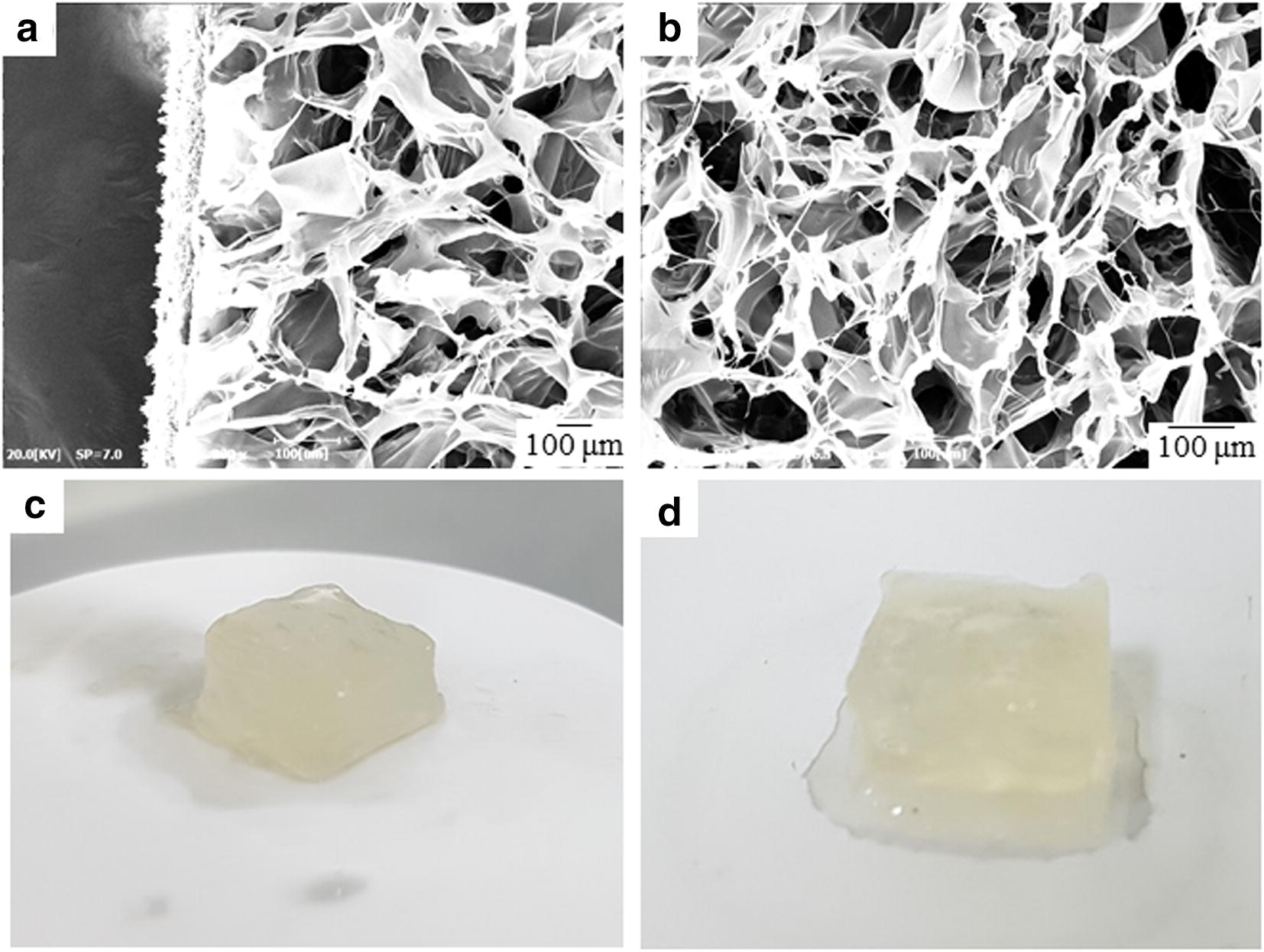
The porous network (**a**, **b**) and optical image (**c**, **d**) of 3D-printed crosslinked HA structure

The 3D-printed nanocomposite hydrogels with spatiotemporal 3D printing construction were visualized both optically and with SEM. The HA nanocomposite hydrogel revealed that the 3D-dimensional porous networked structure was uniformly distributed with an average pore size of 128 μm (Fig. 4a, b). Reproducibility in size, shape, and fidelity of structure are shown as optical images in Fig. 4c, d.

**Fig. 5:**
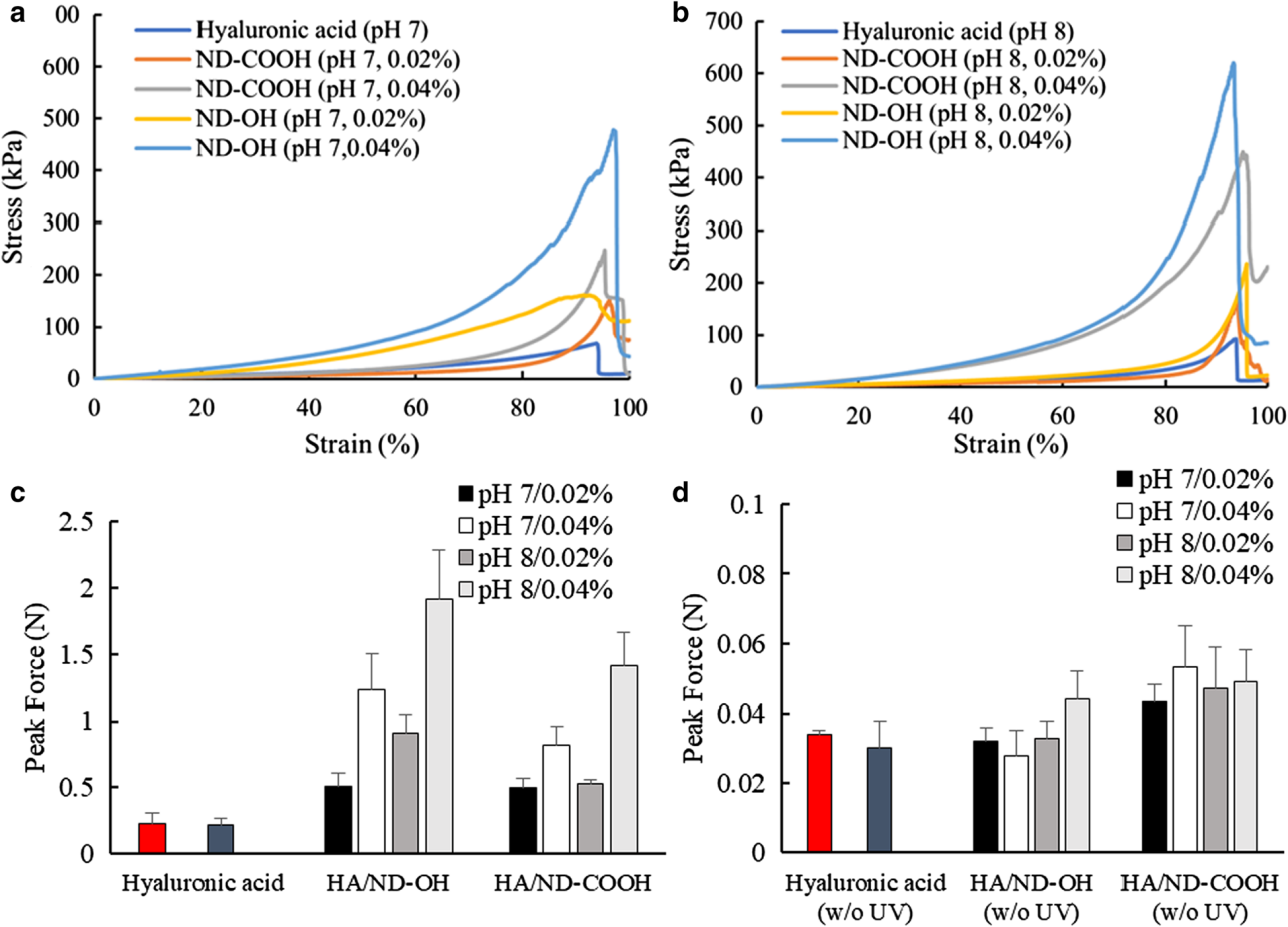
Compressive stress–strain curves of the HA nanocomposite hydrogels with ND-COOH or ND-OH at **a** pH 7 and **b** pH 8. **c** Gel breaking compressive peak force of the HA structures at pH 7 or 8 and surface functionalized NDs [HA without ND at pH 7 (red), pH 8 (blue)]. **d** Compressive peak force of HA structure without post-UV exposure (HA without ND at pH 7 (red), pH 8 (dark gray)

The compressive mechanical stress of nanocomposite hydrogels containing ND-COOH and ND-OH were compared with HA only hydrogels. Nanocomposite hydrogel precursors of HA/ND-COOH and HA/ND-OH were prepared using either 0.02 wt% or 0.04 wt% ND with 2 wt% of MeHA and printed using predetermined printing conditions. The highest level of compressive stress was observed in the vicinity of 90–93% of the compressive strain, and a gel breakage was observed when strain was higher than 90% of the strain. The 3D structure of HA/ND-COOH (0.02 wt%) and HA/ND-OH(0.02 wt%) nanocomposite hydrogels showed maximum compressive stresses of 152.82 kPa, and 161.59 kPa at pH 7, showing 2.2- and 2.35-fold, respectively, higher than the HA only hydrogels (68.72 kPa) (Fig. 5a). With the addition of higher composition of ND-COOH (0.04 wt%), the compressive stress was increased by 163%, compared to that of the lower composition ND-COOH hydrogel (0.02 wt%). Moreover, the 0.04 wt% composite ratio of ND-OH resulted in the stiffest gradient and dramatically enhanced peak compressive force of 296% (478.03 kPa), compared to that of hydrogel for ND-COOH (0.02 wt%). Similar compressive stress was observed for HA/ND-COOH and HA/ND-OH nanocomposite hydrogels when processed at pH 8 (Fig. 5b). HA nanocomposite hydrogels with ND-OH at pH 8 showed an increase of 1.40-fold (236.18 kPa) and 1.37-fold (616.72 kPa) compressive stress at the composition of 0.02 wt% and 0.04 wt%, respectively, compared to those of ND-COOH [168.31 kPa (0.02 wt% ND-COOH), 449.59 kPa (0.04 wt% ND-COOH)] at the same pH 8. Moreover, the compressive stress of HA/ND-OH (0.04 wt%) at pH 8 was mechanically enhanced 1.29-fold, compared to that of HA/ND-OH (0.04 wt%) at pH 7.

The results indicate that 3D printed HA hydrogels filled with hydrophilic surface functionalized nanodiamond boost mechanical properties even at low weight ratios of ND. The enhanced mechanical properties can be attributed to the high surface-to-volume ratio and hydrophilic ND surface which generated sufficient nanophase distribution within hydrogel matrix [50].

The comparison of pH dependence and the effect of surface functional groups are also apparent in the gel peak breaking force. 3D-printed HA/ND nanocomposite hydrogels with (Fig. 5c) and without (Fig. 5d) post-UV exposure were used to investigate the impact of pH on mechanical integrity. Both HA nanocomposite hydrogels containing ND-COOH or ND-OH showed higher peak forces at pH 8, compared to the same nanocomposite hydrogels prepared at pH 7. The difference in pH at the stage of nanocomposite hydrogel precursor resulted in the high peak force 0.905 N of HA/ND-OH (0.02 wt%) at pH 8, compared to 0.511 N of HA/ND-OH (0.02 wt%) at pH 7, revealing a 177% boost in peak force. pH dependence became even more apparent when the composite amount was increased to 0.04 wt%, which showed the enhancement of the peak force with an increase in pH. The peak force 1.920 N of HA/ND-OH (0.04 wt%) at pH 8 showed 155% amplified peak force, compared to 1.236 N of HA/ND-OH (0.04 wt%) at pH 7. The boosted mechanical properties of HA/ND-COOH showed the same pH dependent tendencies. Without post-UV exposure, the gel peak breaking force of the nanocomposite hydrogels at varied pH was not critically different from any sample when compared to HA only hydrogels (Fig. 5d). A change in the gel peak breaking force post-UV crosslinking confirmed that NDs within an HA matrix were spread out in a nanophase, thus concerting molecular environments that promoted interactions between the ND and HA. Hydrogels containing ND-OH showed the best performance at pH 8 and pH 7, compared to those with ND-COOH. The agglomerate stability of the ND varied depending on the pH and surface functionality in the aqueous dispersion [42]. State of ink precursors was a viscous dispersion of HA and ND before extrusion processing, and settled 3D firm hydrogels allowed for post-UV exposure, allowing for stable ND dispersion without confinement at local foci. Agglomerates of hydroxylated NDs are stable at both pH 7 and 8, while similar agglomerates of carboxylate ND are critically enhanced at pH 8 due to the deprotonation of the carboxyl group resulting in a hydrophilic surface. These intrinsic properties of NDs affect the distribution within the HA matrix, intermolecular electrostatic interaction, and hydrogel bonding through physical contact of gel network. Without chemical crosslinking, the physical network created by the addition of ND-COOH or ND-OH was able to achieve the mechanically stabilized 3D-printed hydrogel construction.

## Conclusion

3D printing nanocomposite hydrogels composed of HA/ND achieved mechanically enforced compressive stress when compared with HA hydrogels. The addition of ND at a low weight ratio of 0.02 wt% increased the compressive force and gel breaking point. Surface functionalization of ND with carboxyl and hydroxyl group provided a tunable buffering environment and facilitated interaction with long HA chains at the molecular level, resulting in pH-dependent control of mechanical properties. The ND-OH nanophase, which was stable over a range of pH, improved mechanical stress compared to ND-COOH, indicating that the stability of inorganic compositing nanophases in an aqueous state is a critical parameter that affects mechanical properties of 3D-printed nanocomposite hydrogels. Herein, we have applied filament-based processing and layer-based deposition at the microscale, resulting in newly generated mechanical properties of nanocomposite hydrogels. Fine surface tuning of inorganic ND nanophases along with 3D-printed construction resulted in pH-dependent control over the mechanical properties of HA hydrogels, opening new potentials of metastatic soft and strong nanocomposite materials.
